# Achieving ≥70% of energy requirements during early enteral nutrition and short-term inflammatory–metabolic profiles in critically ill patients with sepsis: a prospective cohort study

**DOI:** 10.3389/fnut.2026.1878377

**Published:** 2026-07-29

**Authors:** Hakan Toğuç, Ahmet Mustafa Altuntaş, Mehmet Çavdar

**Affiliations:** 1 Department of Nutrition and Dietetics, Faculty of Health Sciences, Inonu University, Malatya, Türkiye; 2 Department of Nutrition and Dietetics, Mehmet Akif Inan Training and Research Hospital, Şanlıurfa, Türkiye

**Keywords:** early enteral nutrition, energy adequacy, inflammation, sepsis, biochemical parameters

## Abstract

**Background:**

Sepsis is characterized by dysregulated inflammation, profound metabolic stress, and a high risk of protein–energy malnutrition in critically ill patients. This study examined the association between achieving ≥70% of estimated energy requirements during early enteral nutrition and short-term inflammatory–metabolic profiles in patients with sepsis.

**Methods:**

This prospective observational cohort study was conducted between November 2024 and May 2025 in the intensive care unit of a state hospital in Turkey. A total of 201 patients with sepsis receiving enteral nutrition were classified according to whether they achieved <70% or ≥70% of estimated energy requirements during the first 7 days. Biochemical and inflammatory parameters were assessed at baseline and on Day 7, and prognostic nutritional indices, including the Prognostic-Nutritional-Index (PNI) and C-reactive-protein–albumin–lymphocyte (CALLY) index, were calculated. Between-group changes and adjusted day 7 outcomes were evaluated using appropriate comparative tests and ANCOVA models adjusted for baseline values, age, sex, BMI, and NRS-2002.

**Results:**

The ≥70% energy adequacy group showed significant within-group reductions in CRP, urea, and creatinine by Day 7 (all *p* < 0.001). Between-group comparisons of days 7**–**0 changes showed significant differences for Δurea (*p* < 0.001), Δlymphocyte (*p* < 0.001), Δmonocyte (*p* < 0.001), and ΔPNI (*p* = 0.007), whereas ΔCRP (*p* = 0.083) and Δcreatinine (*p* = 0.156) did not differ significantly. In adjusted ANCOVA models, achieving ≥70% energy adequacy was associated with approximately 30% lower day 7 CRP values [exp(*β*) = 0.70, 95% CI:0.53–0.92, *p* = 0.011] and 18% lower day 7 urea values [exp(β) = 0.82, 95% CI:0.71–0.95, *p* = 0.007]. Albumin showed a borderline positive association (*B* = 1.14, *p* = 0.050), whereas creatinine, NLR, leukocyte count, PNI, and CALLY were not significantly associated with energy adequacy.

**Conclusion:**

The between-group difference in the prespecified change in CRP was not statistically significant. However, secondary adjusted analyses showed lower Day 7 CRP and urea values in patients achieving ≥70% energy adequacy. These findings should be considered exploratory and interpreted cautiously because of the observational design.

## 1 Introduction

Sepsis is a life-threatening condition caused by a dysregulated host response to infection and remains a major global public health problem with substantial morbidity, mortality, and healthcare burden. It causes millions of cases and high mortality rates worldwide each year, placing a significant burden on healthcare systems (1, 2). The pathophysiology of sepsis is a complex process involving a systemic inflammatory response syndrome triggered by the uncontrolled release of pro-inflammatory and anti-inflammatory cytokines. This “cytokine storm” leads to widespread endothelial damage, microcirculatory dysfunction, mitochondrial dysfunction, and ultimately multiple organ failure (3, 4). The high global burden of sepsis and its long-term mortality/incidence rates make the optimization of supportive therapies, as well as early diagnosis and treatment strategies, particularly in intensive care units, critical (5).

The hypermetabolic and hypercatabolic response to sepsis promotes insulin resistance, accelerated proteolysis, loss of lean body mass, and reduced nutrient intake, thereby increasing the risk of disease-related malnutrition. This relationship is bidirectional, as sepsis may worsen nutritional status, whereas malnutrition may impair immune function and reduce tolerance to infection-related metabolic stress (6, 7). Recent studies support the prognostic relevance of nutrition-related indices in sepsis. Toscano et al. reported that nutrition- and inflammation-based scores, particularly the modified Nutrition Risk in Critically Ill score, were associated with in-hospital mortality in patients with sepsis or septic shock (8). Similarly, a multicenter study of 9,763 patients with sepsis showed that moderate and severe malnutrition classified using the CONUT score and PNI was associated with increased 30-day mortality (9). Evidence from broader hospitalized populations also supports the clinical relevance of malnutrition-related assessment (10). Therefore, early nutritional risk assessment and appropriate nutritional support are integral components of sepsis management.

Enteral nutrition is considered the physiological route compared to parenteral nutrition. Early enteral nutrition (initiated within the first 24–48 h) offers theoretical advantages by preserving intestinal villus structure, maintaining mucosal barrier function, and reducing bacterial translocation (11). Furthermore, it contributes to preventing the concept of “gut-associated sepsis” and modulating hepatic protein synthesis (12). International guidelines recommend early enteral nutrition in hemodynamically stable critically ill patients (13). However, the optimal nutritional strategy remains controversial, particularly in hemodynamically sensitive populations such as patients with severe sepsis and septic shock. In these patients, splanchnic perfusion may be reduced, and early aggressive enteral nutrition may increase the risk of intestinal ischemia or aspiration (14).

Studies on the effect of enteral nutrition on clinical outcomes [mortality, intensive care unit (ICU) length of stay] in septic patients have reported conflicting results (15, 16). Therefore, the research focus has shifted to intermediate outcomes that may help us better understand the potential beneficial mechanisms of enteral nutrition. Inflammatory markers [C-reactive protein (CRP), interleukin-6 (IL-6), tumor necrosis factor-alpha (TNF-α)] and biochemical nutritional parameters (albumin, prealbumin, lymphocyte count) are critical for monitoring sepsis severity, prognosis, and nutritional status dynamics (17–19). The modulatory effect on these parameters reflects enteral nutrition’s impact on systemic inflammation and metabolic imbalance.

In the literature, while there are comprehensive studies on the effects of early enteral feeding (EEF) in critically ill patients in general, prospective cohort studies focusing on the effects of energy adequacy on the course of inflammatory and biochemical parameters in patients with sepsis are limited. Current studies are mostly retrospective in design or include heterogeneous patient groups.

The aim of this prospective cohort study is to evaluate the relationship between energy adequacy in early enteral nutrition and the course of inflammatory and biochemical parameters during the first 7 days in critically ill patients with sepsis. The pre-specified hypothesis of the study is that in patients meeting at least 70% of their energy requirements, a more pronounced reduction in inflammatory markers—particularly C-reactive protein—and a more favorable change in nutrition-related biochemical indicators will be observed compared to patients falling below this level. By clarifying this association, the study seeks to contribute to a better understanding of the potential clinical relevance of early energy adequacy in sepsis and to inform future nutrition strategies for this high-risk population.

## 2 Materials and methods

This prospective observational cohort study was conducted in the adult intensive care unit of the Mehmet Akif İnan Training and Research Hospital, Şanlıurfa, Turkey, between November 2024 and May 2025. Enteral feeding practices were planned in accordance with clinical routine; researchers did not intervene in the selection of feeding formula, timing of initiation, or dosage targets. Sepsis was diagnosed by the attending intensive care physician according to the Sepsis-3 criteria, defined as suspected or documented infection accompanied by an acute increase of at least 2 points in the Sequential Organ Failure Assessment score. Individual SOFA scores were not retained in the study database and were therefore unavailable for severity-adjusted analyses.

Inclusion criteria were a diagnosis of sepsis, having been in the intensive care unit for more than 24 h after the start of treatment, and being able to receive enteral nutrition. Exclusion criteria included chronic liver disease, chronic renal failure, acute kidney failure, active cancer, and parenteral nutrition.

**Figure 1:**
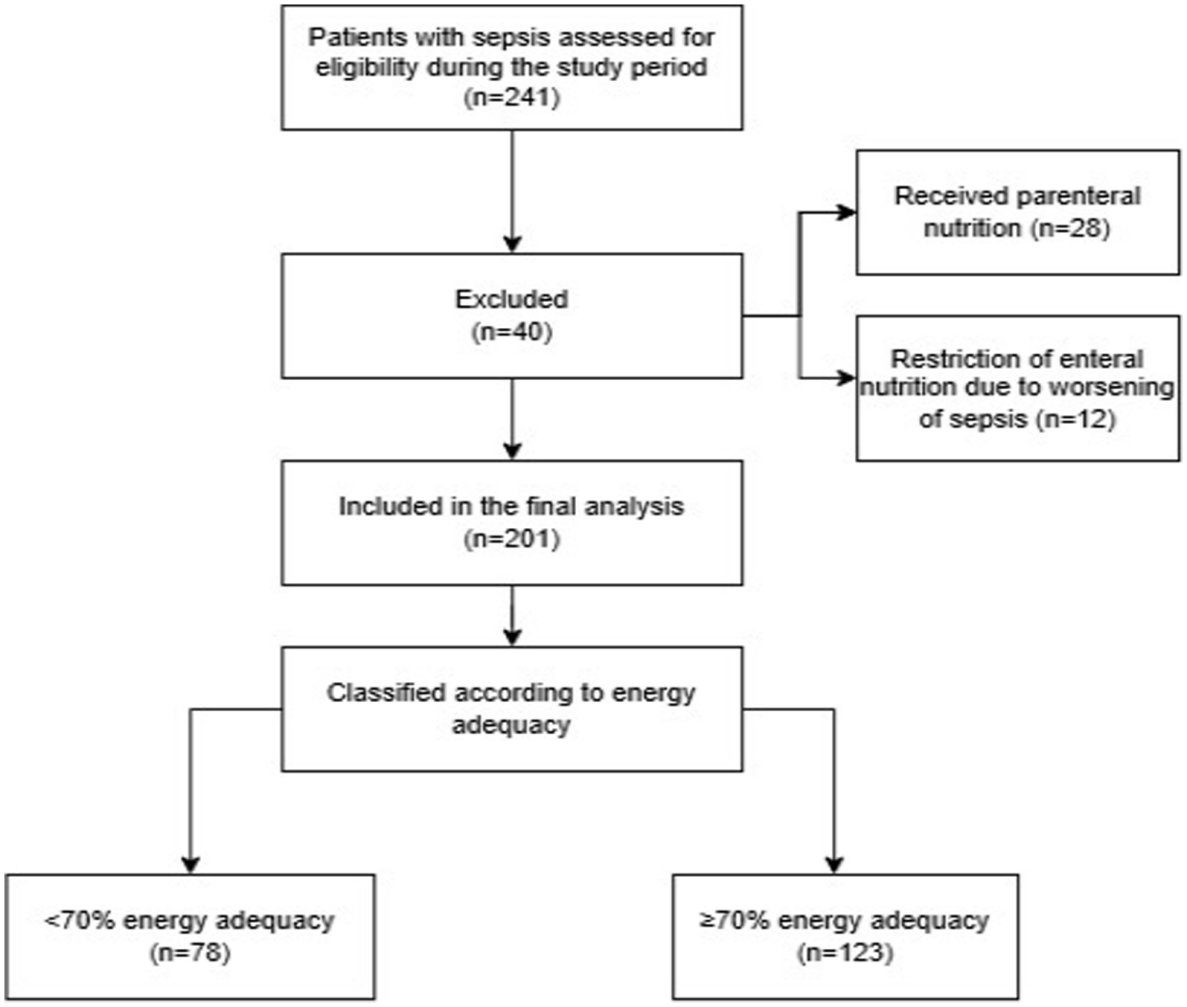
Flow chart.

All patients admitted to the ICU during the study period were consecutively screened, and those meeting the eligibility criteria were enrolled in the study. In addition, a supplementary sample size calculation was performed using the day 7 CRP data reported by Hosny et al. in a comparable early sepsis population; with a two-sided alpha of 0.05 and 90% power, the minimum required sample size was 49 patients per group, which increased to 54 patients per group after allowing for a 10% attrition rate (20). During the study period, 241 patients with sepsis were assessed for eligibility; 12 patients who required discontinuation of enteral nutrition within the first week due to clinical deterioration and 28 patients receiving parenteral nutrition were excluded, and data from 201 patients were included in the final analysis (Figure 1).

### 2.1 Research hypothesis and outcomes

The predefined hypothesis of this study is that sepsis patients who achieve at least 70% of their energy requirements during the early enteral feeding phase will demonstrate more favorable inflammatory and biochemical changes within the first 7 days compared to patients whose energy adequacy remains below 70%.

The primary outcome of the study was the change in serum CRP levels from baseline to day 7. The secondary outcomes were the changes in albumin, urea, creatinine, leukocytes, neutrophils, lymphocytes, monocytes, neutrophil-to-lymphocyte ratio (NLR), Prognostic Nutrition Index (PNI) and C-reactive protein-albumin-lymphocyte (CALLY) indices between baseline and day 7, as well as the adjusted results on day 7.

### 2.2 Data collection and variables

Demographic and anthropometric variables included age, sex, height, weight, and BMI. Clinical variables included the major diagnoses documented at ICU admission and the available treatment-related information. Nutritional variables included Nutritional Risk Screening 2002 (NRS-2002) score, enteral formula volume, energy and protein intake, calculated requirements, adequacy percentages, and energy and protein deficits. Laboratory variables were obtained at baseline and on day 7 and included glucose, albumin, CRP, hemoglobin, urea, creatinine, and leukocyte subtypes. Major clinical diagnoses documented at ICU admission were extracted from patient records. Because the available data did not permit reliable classification of these conditions as the source of sepsis, the primary reason for ICU admission, or a comorbidity, they were reported descriptively and were not treated as uniform etiological categories. Sepsis treatment was managed by the attending intensive care team according to routine clinical practice. No intravenous albumin supplementation was administered during the seven-day observation period. Detailed data on antimicrobial regimens, corticosteroid exposure, and organ-support strategies were not systematically collected.

Biochemical and inflammatory data relating to primary and secondary endpoints were obtained from patient records at baseline (day 0) and on day 7. These parameters included glucose, albumin, CRP, hemoglobin, urea, creatinine, leukocytes, neutrophils, lymphocytes, monocytes, eosinophils and basophils. In addition, the Nutritional Risk Score-2002 (NRS-2002) was recorded at baseline; the PNI and the CALLY indices were calculated using data from days 0 and 7.

NRS-2002: Nutritional Risk Screening 2002 is an evidence-based screening tool developed by the European Society for Clinical Nutrition and Metabolism (ESPEN) for the early detection of malnutrition risk in hospitalized individuals. It is widely used in assessing nutritional status in adults. This scale generates a total risk score by evaluating criteria such as recent weight loss, reduced food intake, BMI, and the metabolic stress level of the current illness. Two main components are assessed in the NRS-2002 calculation: nutritional status (weight loss, BMI, food intake) and disease severity. Both components are scored between 0 and 3; an additional point is awarded to patients aged 70 years and older, and individuals with a total score ≥3 are considered to be at nutritional risk (21). NRS-2002 was administered by the clinical dietitian who was also a study investigator within 48 h of admission to the intensive care unit. In patients unable to communicate, information regarding recent weight loss and reduced food intake was obtained from medical records, nursing documentation, and/or legally authorized representatives.

PNI: PNI is a composite indicator reflecting both nutritional and immune status. It is commonly used in the assessment of surgical risk and prediction of clinical prognosis. PNI is calculated using the following formula: serum albumin (g/L) + 5 × total lymphocyte count (10^9^/L). A PNI score below 45 is considered indicative of malnutrition (22).

CALLY: In this study, the CRP–albumin–lymphocyte (CALLY) index was calculated to integrate inflammation (C-reactive protein; CRP), nutritional/visceral protein status (serum albumin), and immune cellular response (absolute lymphocyte count) into a single composite measure. The CALLY index was first defined in oncology practice as a composite biomarker associated with prognosis, and studies have subsequently been published linking it to risk stratification and clinical outcomes in the context of sepsis and critical illness (23). Given the limited validation in septic ICU populations, CALLY was evaluated in the present study as an exploratory composite index rather than a validated sepsis-specific prognostic tool.


CALLY=(Albumin×Absolute lymphocyte count)/CRP

### 2.3 Nutritional information

As part of the study, the enteral formulations taken by patients over a 7-day period were recorded by the researcher, and the total score was divided by 7 to calculate the average daily energy and protein intake. Accordingly, the participants’ energy requirement was calculated as 25 kcal/kg/day and their protein requirement as 1.3 g/kg/day. The calculation was based on the patients’ ideal body weight (IBW). The Devine (metric) IBW formula, commonly used in clinical studies, was used to calculate the “ideal body weight” (based on height and gender data) (24–26):


Male:IBW=50+0.9×(Height(cm)−152)

Female:IBW=45.5+0.9×(Height(cm)−152)

### 2.4 Statistical evaluation

The data obtained from the study were analyzed using the SPSS 25.0 software package. Continuous variables were assessed for normal distribution using the Kolmogorov–Smirnov test; they were reported as median (IQR) according to their distribution characteristics. Categorical variables were summarized as counts and percentages. Energy adequacy was classified as <70% and ≥70% based on the calculated energy requirement (27). For intergroup comparisons, the independent samples *t*-test and Mann–Whitney U test were used for continuous variables, and appropriate chi-square tests were used for categorical variables. Parametric/non-parametric approaches were selected according to the distribution for analyses of changes from days 0 to 7. Spearman’s rank correlation analysis was used to examine associations between continuous energy and protein adequacy percentages and changes in biochemical and inflammatory parameters. Adjusted analyses were performed using an analysis of covariance approach. For each model, the day 7 value was entered as the dependent variable, energy-adequacy group as the main independent variable, and the corresponding baseline value, age, sex, BMI, and NRS-2002 score as covariates. Covariates were selected *a priori* based on clinical relevance and observed baseline imbalances rather than through an automated stepwise procedure. CRP, urea, creatinine, and NLR were natural-log transformed because of their right-skewed distributions. Effects for log-transformed outcomes were reported as exponentiated regression coefficients [exp(*β*)], representing adjusted ratios, whereas untransformed outcomes were reported as unstandardized regression coefficients (B), with 95% confidence intervals. Illness-severity scores and organ-support variables were not included because these data were not systematically available. No adjustment for multiple comparisons was applied to the secondary analyses; therefore, these findings were considered exploratory. All tests were two-sided, and *p* < 0.05 was considered statistically significant.

## 3 Results

General information regarding the participants’ baseline energy adequacy is presented in Table 1. The median age of the 201 sepsis patients in intensive care included in the study was 73.0; 46.3% were female and 53.7% were male. While no significant differences were observed between groups in terms of age (*p* = 0.594) and NRS-2002 scores (*p* = 0.357) with regard to energy adequacy, significant differences were observed in anthropometric measurements such as height (*p* = 0.001), weight (*p* = 0.003) and BMI (*p* < 0.001). The proportion of women was higher in the group with energy adequacy of 70% or above (*p* < 0.001). While there were no differences between the groups in terms of baseline laboratory parameters such as glucose, urea, creatinine, albumin, CRP and hemoglobin, there were significant differences in inflammatory parameters (Lymphocyte, NLR, PNI and CALLY) (*p* = 0.003; *p* < 0.001; *p* = 0.004; *p* = 0.004 respectively). The ≥70% energy-adequacy group had higher baseline lymphocyte, PNI, and CALLY values and lower NLR values, suggesting a more favorable baseline immune-nutritional profile. No statistically significant between-group differences were observed in the major clinical diagnoses documented at ICU admission.

**Table 1:** Classification of participants’ general information according to their energy adequacy intake.

Variable	Total	*p*	<70% (Energy)	≥70% (Energy)
Demographic/Clinical	Med [IQR]		Med [IQR]	Med [IQR]
Age (years)	73 [59–80]	0.594	72 [57.75–80.75]	73 [59.5–80]
Height (cm)	168 [160–175]	0.001	171 [165–175]	165 [158.5–173.5]
Weight (kg)	75 [65–85]	0.003	70 [61.25–80]	76 [68–85]
BMI (kg/m^2^)	25.88 [23.44–29.97]	<0.001	24.36 [22.15–26.74]	27.76 [24.49–31.22]
NRS-2002	4 [2–5]	0.357	4 [2–5]	4 [1–5]

Gender	*n* (%)		*n* (%)	*n* (%)
Female	93 (46.3%)	<0.001	20 (25.6%)	73 (59.3%)
Male	108 (53.7%)	<0.001	58 (74.4%)	50 (40.7%)

Major clinical diagnoses	*n* (%)		*n* (%)	*n* (%)
Pneumonia	86 (42.8%)	1.000	33 (42.3%)	53 (43.1%)
Cerebrovascular diseases	84 (41.8%)	0.252	37 (47.4%)	47 (38.2%)
Cardiac arrest	15 (7.5%)	0.067	2 (2.6%)	13 (10.6%)

Initial biochemical parameters	Med [IQR]		Med [IQR]	Med [IQR]
Glucose (mg/dL)	132 [112–182]	0.716	128 [106.5–210]	132 [113–177.5]
Urea (mg/dL)	50.7 [37.5–80]	0.763	48.85 [36.83–85.3]	53 [37.65–78.3]
Creatinine (mg/dL)	0.75 [0.54–1.16]	0.056	0.82 [0.57–1.42]	0.7 [0.53–1.06]
Albumin (g/L)	29.1 [24.5–32]	0.277	27.9 [23.62–32.15]	29.6 [25.6–31.7]
CRP (mg/L)	94.8 [64.08–165.12]	0.148	98.2 [73.38–164]	92.93 [40.72–165.5]
Hemoglobin (g/dL)	10 [9.1–11.5]	0.719	10 [8.83–11]	10.1 [9.1–11.6]
Leukocyte (×10^9^/L)	11.48 [8.86–15.93]	0.587	11.72 [8.3–17.95]	11.4 [9.14–15.71]
Neutrophil (×10^9^/L)	9.44 [6.69–14.08]	0.132	10.34 [6.84–15.81]	9.32 [6.49–13.68]
Lymphocyte (×10^9^/L)	1.08 [0.74–1.68]	0.003	0.96 [0.67–1.39]	1.19 [0.88–1.83]
Monocyte (×10^9^/L)	0.69 [0.48–0.97]	0.084	0.62 [0.41–0.97]	0.7 [0.53–0.96]
Eosinophil (×10^9^/L)	0.04 [0–0.17]	0.840	0.07 [0–0.16]	0.04 [0–0.18]
Basophil (×10^9^/L)	0.01 [0–0.01]	0.345	0.01 [0–0.01]	0.01 [0.01–0.01]
NLR	8.33 [5.17–14.64]	<0.001	10.53 [6.67–21.59]	7.01 [4.75–12.81]
PNI	40.5 [35.4–46.2]	0.004	38.6 [32.97–43.45]	41.2 [36.6–47.5]
CALLY	0.27 [0.16–0.64]	0.004	0.24 [0.14–0.37]	0.36 [0.17–0.82]

CRP, C-Reactive Protein; NLR, Neutrophil-to-Lymphocyte Ratio; PNI, Prognostic Nutritional Index; CALLY index, C-reactive protein–albumin–lymphocyte Index, *p* < 0.05.

Table 2 presents the nutritional goals and intake status of participants according to their energy adequacy. Patients with energy adequacy <70% had a higher ideal weight (*p* < 0.001) and calculated energy requirement (*p* < 0.001). Similarly, energy and protein intake were also higher in the ≥70% group (*p* < 0.001 for both). In terms of adequacy rates, the medians of energy adequacy and protein adequacy were 85.4 and 83.1%, respectively, in the ≥70% energy adequacy group, while these rates were 56.6 and 57.3%, respectively, in the <70% group (*p* < 0.001 for both). Energy and protein deficiencies were higher in the <70% group (*p* < 0.001).

**Table 2:** Nutritional status of participants according to their energy adequacy intake.

Variable	Total	*p*	<70% (Energy)	≥70% (Energy)
Adequacy classification	*n* (%)		*n* (%)	*n* (%)
Energy <70%	78 (38.8%)		78 (100.0%)	0 (0.0%)
Energy ≥70%	123 (61.2%)		0 (0.0%)	123 (100.0%)

Nutritional targets and intake (7-day average)	Med [IQR]	*p*	Med [IQR]	Med [IQR]
Ideal weight (kg)	63.5 [52.7–70.7]	<0.001	67.1 [61.7–70.7]	57.2 [51.35–68]
Energy requirement (kcal/day)	1,588 [1318–1768]	<0.001	1,677 [1542–1768]	1,430 [1283.5–1700]
Protein requirement (g/day)	82.5 [68.5–91.9]	<0.001	87.2 [80.2–91.9]	74.4 [66.75–88.4]
Energy intake (kcal/day)	1,144 [990–1,334]	<0.001	915 [801–1026.5]	1318.6 [1144–1433.75]
Protein intake (g/day)	59 [51.6–69.87]	<0.001	51.1 [37.7–57.1]	65.3 [56.35–74.89]
Energy (kcal/kg IBW/day)	19.66 [15.5–22.19]	<0.001	14.16 [12.1–15.8]	21.34 [19.91–23.73]
Protein (g/kg IBW/day)	0.98 [0.81–1.14]	<0.001	0.74 [0.64–0.82]	1.08 [0.99–1.25]
Energy adequacy (%)	78.64 [61.98–88.79]	<0.001	56.62 [48.42–63.19]	85.37 [79.62–94.94]
Protein adequacy (%)	75.62 [62.13–87.74]	<0.001	57.27 [49.58–62.83]	83.08 [76.03–96.23]
Energy deficit (kcal/day)	338 [181–615.5]	<0.001	697.5 [565–851.42]	204 [62.9–307.5]
Protein deficit (g/day)	17.9 [9.5–32.9]	<0.001	35.4 [28.52–42.87]	11.7 [2.44–17.49]

Table 3 presents the intra-group changes in biochemical and inflammatory parameters from days 0 to 7, according to energy adequacy. In the group with energy adequacy of 70% or higher, significant decreases were observed in urea, creatinine, albumin, CRP, white blood cell count, neutrophils, monocytes, hemoglobin (*p* < 0.001 for all) and PNI (*p* = 0.002), while an increase in CALLY (*p* < 0.001) was observed, and no significant changes were observed in glucose, lymphocytes, eosinophils, basophils or NLR (*p* > 0.05). In the <70% energy adequacy group, however, significant intragroup changes were observed in creatinine (*p* = 0.002), albumin (*p* < 0.001), lymphocytes (*p* < 0.001), eosinophils (*p* = 0.018), hemoglobin (*p* = 0.001), NLR (*p* < 0.001) and CALLY (*p* = 0.005), while no significant changes were observed in glucose, urea, CRP, leukocytes, neutrophils, monocytes, basophils or PNI.

**Table 3:** Within-group changes in biochemical and inflammatory parameters from days 0 to 7 according to energy adequacy.

Parameter	<70%-day 0 (Median, IQR)	<70%-day 7 (Median, IQR)	<70% *p*	≥70%-day 0 (Median, IQR)	≥70%-day 7 (Median, IQR)	≥70% *p*
Glucose (mg/dL)	128 [106.5–210]	135 [111–193]	0.529	132 [113–177.5]	123 [107.5–163.5]	0.181
Urea (mg/dL)	48.85 [36.83–85.3]	55.4 [37–98.4]	0.265	53 [37.65–78.3]	43 [30.65–61.4]	<0.001
Creatinine (mg/dL)	0.82 [0.57–1.42]	0.69 [0.47–1.27]	0.002	0.7 [0.53–1.06]	0.55 [0.41–0.82]	<0.001
Albumin (g/L)	27.9 [23.62–32.15]	25.1 [23.02–29.4]	<0.001	29.6 [25.6–31.7]	28.1 [24.75–30]	<0.001
CRP (mg/L)	98.2 [73.38–164]	99 [38.5–168]	0.393	92.93 [40.72–165.5]	58.14 [31.64–90.9]	<0.001
Leukocyte (×10^9^/L)	11.72 [8.3–17.95]	11.62 [9.1–15.79]	0.311	11.4 [9.14–15.71]	9.6 [7.94–12.2]	<0.001
Neutrophil (×10^9^/L)	10.34 [6.84–15.81]	9.19 [6.42–13.27]	0.078	9.32 [6.49–13.68]	7.5 [5.43–10.7]	<0.001
Lymphocyte (×10^9^/L)	0.96 [0.67–1.39]	1.24 [0.94–1.56]	<0.001	1.19 [0.88–1.83]	1.3 [0.91–1.64]	0.159
Monocyte (×10^9^/L)	0.62 [0.41–0.97]	0.7 [0.56–0.94]	0.210	0.7 [0.53–0.96]	0.64 [0.41–0.85]	<0.001
Eosinophil (×10^9^/L)	0.07 [0–0.16]	0.14 [0.03–0.27]	0.018	0.04 [0–0.18]	0.1 [0.01–0.2]	0.083
Basophil (×10^9^/L)	0.01 [0–0.01]	0.01 [0.01–0.02]	0.068	0.01 [0.01–0.01]	0.01 [0.01–0.01]	0.962
Hemoglobin (g/dL)	10 [8.83–11]	9.5 [8.62–10.75]	0.001	10.1 [9.1–11.6]	9.5 [8.5–10.7]	<0.001
NLR	10.53 [6.67–21.59]	8.14 [5.63–11]	<0.001	7.01 [4.75–12.81]	6.33 [4.44–11.96]	0.593
PNI	38.6 [32.97–43.45]	37.9 [34.48–43.2]	0.334	41.2 [36.6–47.5]	39.7 [34.55–46.4]	0.002
CALLY	0.24 [0.14–0.37]	0.32 [0.18–0.85]	0.005	0.36 [0.17–0.82]	0.57 [0.22–1.34]	<0.001

CRP, C-Reactive Protein; NLR, Neutrophil-to-Lymphocyte Ratio; PNI, Prognostic Nutritional Index; CALLY index, C-reactive protein–albumin–lymphocyte Index, *p* < 0.05.

Table 4 compares the days 7–0 changes between the two energy adequacy groups. Significant between-group differences were found for Δurea (*p* < 0.001), Δlymphocyte (*p* < 0.001), Δmonocyte (*p* < 0.001), and ΔPNI (*p* = 0.007). Specifically, the ≥70% group showed a more favorable change in urea, whereas the <70% group showed higher positive changes in lymphocyte and monocyte counts and a more favorable change in PNI. No significant between-group differences were observed for Δglucose, Δcreatinine, Δalbumin, ΔCRP, Δleukocyte, Δneutrophil, Δhemoglobin, ΔNLR, or ΔCALLY. Accordingly, the prespecified primary endpoint, defined as the between-group difference in the change in CRP from baseline to day 7, was not statistically significant.

**Table 4:** Between-group comparison of days 7–0 changes in biochemical and inflammatory parameters according to energy adequacy.

Parameter (*Δ*)	<70% Δ (*n* = 78)(Median, IQR)	≥70% Δ (*n* = 123) (Median, IQR)	*p*
Δ Glucose (G7–G0)	3.5 [−26.25 to 37]	−1 [−22.5 to 9]	0.149
Δ Urea (G7–G0)	3.3 [−14.45 to 21.6]	−8.8 [−23.8 to −2]	<0.001
Δ Creatinine (G7–G0)	−0.1 [−0.33 to 0.05]	−0.15 [−0.23 to −0.05]	0.156
Δ Albumin (G7–G0)	−1.6 [−3.95 to 0.8]	−1.6 [−2.83 to 0.2]	0.795
Δ CRP (G7–G0)	−17.6 [−64.39 to 57.38]	−21.62 [−84.4 to −2.25]	0.083
Δ Leukocyte (G7–G0)	−0.63 [−5.46 to 3.1]	−2.33 [−4.34 to 0.1]	0.210
Δ Neutrophil (G7–G0)	−0.8 [−5.37 to 2.17]	−2.18 [−4.21 to −0.01]	0.816
Δ Lymphocyte (G7–G0)	0.24 [−0.07 to 0.56]	−0.14 [−0.41 to 0.23]	<0.001
Δ Monocyte (G7–G0)	0.04 [−0.15 to 0.25]	−0.08 [−0.29 to 0.09]	<0.001
Δ Hemoglobin (G7–G0)	0.02 [−0.02 to 0.1]	0 [−0.03 to 0.09]	0.210
Δ NLR (G7–G0)	0.01 [0–0.03]	0.02 [−0.01 to 0.03]	0.249
Δ PNI	1.2 [−4.31 to 5.1]	−2.4 [−5.2 to 2.25]	0.007
Δ CALLY	0.05 [−0.06 to 0.37]	0.12 [−0.1 to 0.38]	0.999

Δ = Day 7–0, CRP, C-Reactive Protein; NLR, Neutrophil-to-Lymphocyte Ratio; PNI, Prognostic Nutritional Index; CALLY index, C-reactive protein–albumin–lymphocyte Index, *p* < 0.05.

Table 5 reports the relationship between energy adequacy and day 7 results using adjusted analysis. In the adjusted ANCOVA model, achieving ≥70% energy adequacy was associated with lower day 7 CRP and urea values after adjustment for the available covariates. Because illness-severity and organ-support variables were unavailable, these findings should not be interpreted as severity-adjusted or causal effects [exp*β* = 0.70, *p* = 0.011; exp.(β) = 0.82, *p* = 0.007]. However, no significant relationship was found for other biochemical parameters (creatinine, NLR) and prognostic nutritional parameters (PNI, CALLY) (*p* > 0.05). Because illness-severity and organ-support variables were unavailable, these findings should not be interpreted as severity-adjusted or causal effects.

**Table 5:** Adjusted association between energy-adequacy group and day 7 biochemical and inflammatory outcomes.

Result (day 7)	Adjusted effect estimate	95% CI	*p*
CRP (mg/L)	exp(β) = 0.70	0.53–0.92	0.011
Albumin (g/L)	*B* = 1.14	0.00–2.27	0.050
Creatinine (mg/dL)	exp(β) = 0.82	0.65–1.04	0.106
Urea (mg/dL)	exp(β) = 0.82	0.71–0.95	0.007
NLR	exp(β) = 1.08	0.87–1.33	0.500
Leukocyte (×10^9^/L)	*B* = −1.34	−2.88 to 0.20	0.088
PNI	*B* = 5.90	−6.15 to 17.96	0.337
CALLY	*B* = 0.33	−0.22 to 0.88	0.243

Models were fitted using an ANCOVA approach. Day 7 values were entered as dependent variables, energy adequacy group was entered as the main independent variable, and baseline values, age, sex, BMI, and NRS-2002 were included as covariates. The <70% energy adequacy group was used as the reference category. For log-transformed outcomes, adjusted effects are presented as exp.(β); for non-log-transformed outcomes, adjusted effects are presented as unstandardized regression coefficients (B). Illness-severity scores and organ-support variables were unavailable and were therefore not included in these models.

CRPm C-Reactive Protein, Neutrophil-to-Lymphocyte Ratio; PNIm Prognostic Nutritional Index; CALLY index, C-reactive protein–albumin–lymphocyte Index; BMI, Body Mass Index; NRS-2002: Nutritional Risk Screening 2002, *p* < 0.05.

Table 6 shows the relationship between energy and protein adequacy and inflammatory/biochemical changes. In adjusted ANCOVA models, achieving ≥70% energy adequacy was associated with lower Day 7 CRP and urea levels after adjustment for baseline values, age, sex, BMI, and NRS-2002 (*p* = 0.011). Specifically, the ≥70% energy adequacy group had approximately 30% lower Day 7 CRP values and 18% lower Day 7 urea values compared with the <70% group (*p* = 0.007). Albumin showed a borderline positive association (*p* = 0.050), whereas creatinine, NLR, leukocyte count, PNI, and CALLY were not significantly associated with energy adequacy group (*p* > 0.05).

**Table 6:** Relationship between energy and protein sufficiency (%) and inflammatory/biochemical changes (Δ = Day7 − Day0).

Change (*Δ*)	Energy adequacy *ρ*	Energy p	Protein adequacyρ *ρ*	Protein *p*
ΔCRP (G7 − G0)	−0.163	0.022	−0.152	0.032
ΔAlbumin (G7 − G0)	0.024	0.731	−0.028	0.696
ΔNLR (G7 − G0)	0.169	0.017	0.120	0.089
ΔCreatinine (G7 − G0)	−0.137	0.052	−0.104	0.142
ΔUrea (G7 − G0)	−0.275	<0.001	−0.286	<0.001
ΔWBC (G7 − G0)	−0.006	0.933	−0.077	0.278
ΔPNI (G7 − G0)	−0.067	0.346	−0.103	0.146
ΔCALLY (G7 − G0)	0.046	0.514	0.064	0.368

ρ = Spearman correlation coefficient, Δ = Day 7 − Day 0, NLR, Neutrophil-to-Lymphocyte Ratio; PNI, Prognostic Nutritional Index; CALLY index, C-reactive protein–albumin–lymphocyte Index, *p* < 0.05.

Table 6 presents the correlations between continuous energy and protein adequacy percentages and changes in inflammatory and biochemical parameters. Energy and protein adequacy were weakly inversely correlated with ΔCRP and Δurea (*ρ* = −0.163, *p* = 0.022; ρ = −0.152, *p* = 0.032; ρ = −0.275, *p* < 0.001; ρ = −0.286, *p* < 0.001, respectively). Energy adequacy was also weakly positively correlated with ΔNLR, whereas no significant correlations were observed for Δalbumin, Δleukocyte count, ΔPNI, or ΔCALLY (*p* > 0.05).

## 4 Discussion

This prospective cohort study investigated the relationship between early-stage energy adequacy provided via the enteral route in sepsis patients monitored in intensive care and inflammatory response, key biochemical indicators, and prognostic nutrition indices. Participants were classified according to whether their target energy requirement was met at <70% or ≥70% during the first 7 days; patterns of change in CRP, leukocyte subpopulations, NLR, urea, creatinine, albumin, and composite indicators such as PNI and CALLY were evaluated throughout follow-up. These findings indicate an association between nutritional delivery and short-term inflammatory–metabolic profiles; however, causality cannot be inferred.

Although CRP decreased significantly within the ≥70% energy-adequacy group, the between-group difference in ΔCRP was not statistically significant. Therefore, the prespecified primary endpoint was not met. Nevertheless, in the secondary ANCOVA analysis adjusted for baseline CRP and available covariates, the ≥70% group had lower Day 7 CRP values. This adjusted association should be interpreted cautiously and does not establish a causal anti-inflammatory effect of higher energy delivery. Given that CRP is a widely used marker of inflammatory burden and prognosis in sepsis (28), this finding may suggest a more favorable short-term inflammatory trajectory in patients achieving higher early energy adequacy. A possible explanation is that early enteral nutrition may help preserve intestinal barrier function and modulate mucosal immune responses, thereby potentially limiting secondary immune activation. This interpretation is biologically plausible and is supported by studies emphasizing the role of gut barrier dysfunction and bacterial translocation in the pathophysiology of sepsis and septic shock (29, 30). Nevertheless, the observed association may partly reflect differences in illness severity and early clinical course. Less severely ill, hemodynamically stable, or more enterally tolerant patients may have been more likely both to achieve higher energy adequacy and to show favorable biomarker changes. Because SOFA and APACHE II scores, septic shock status, vasopressor use, mechanical ventilation, and renal replacement therapy were unavailable, residual confounding and reverse causation cannot be excluded.

Baseline differences in BMI, lymphocyte count, NLR, PNI, and CALLY further indicate that the ≥70% group may have had a more favorable initial anthropometric and immune-nutritional profile. Although BMI and the corresponding baseline value of each outcome were included in the adjusted models, these adjustments may not fully account for differences in baseline prognosis or unmeasured clinical severity. Residual confounding therefore remains possible. Compared to the literature, observational/secondary analyses exist suggesting that early enteral feeding is associated with better outcomes in patients with sepsis. For example, Jiang et al. reported that early enteral feeding in patients with sepsis may be associated with prognosis (15). Similarly, early enteral feeding has been shown to be associated with better clinical outcomes in critically ill patients receiving vasopressors and mechanical ventilation (31). However, studies in the literature on this topic are heterogeneous: the NUTRIREA-2 study suggested that early enteral approaches in patients with septic shock should be balanced with specific complication profiles (14). Within this framework, the findings of this research reinforce that approaching energy targets more closely, with appropriate patient selection and close monitoring of tolerance, may provide meaningful advantages at the biomarker level. The higher BMI in the ≥70% group may partly reflect the method used to estimate energy requirements. Because requirements were calculated using ideal body weight, the higher proportion of women, shorter height, and lower ideal body weight in this group resulted in lower estimated energy targets, which may have facilitated achievement of the 70% threshold despite higher actual body weight and BMI. Therefore, higher BMI should not be interpreted as a biological explanation for the more favorable biomarker profile.

In this study, patients achieving ≥70% of their energy requirements showed a more favorable short-term urea trajectory, and energy adequacy was associated with lower day 7 urea values in the adjusted analysis. However, this finding should not be interpreted as direct evidence of improved renal function. Serum urea is a nonspecific marker influenced by protein intake, endogenous catabolism, anabolic resistance, renal clearance, and fluid balance. The EFFORT Protein trial found no overall clinical benefit of a very high protein strategy and suggested possible harm in patients with acute kidney injury or greater illness severity (32, 33). However, protein delivery in the present cohort was substantially lower, limiting direct comparison. Because detailed data on acute kidney injury, SOFA score, and renal replacement therapy were unavailable, these factors may have confounded the observed urea associations. Furthermore, the negative correlations between energy and protein adequacy and Δurea may reflect differences in metabolic course rather than a direct nutritional effect. Previous studies have similarly suggested that nitrogen balance and urea-related measures are influenced by both nutritional delivery and disease-related catabolism and should be regarded as complementary metabolic indicators rather than standalone markers of renal recovery or prognosis (31, 32). Therefore, the present findings should be interpreted as an association between nutritional adequacy and short-term urea-related biochemical trajectory rather than evidence of improved kidney function.

In this study, the increase in the CALLY index by day 7 in the ≥70% energy adequacy group may suggest a more favorable composite profile across CRP, albumin, and lymphocyte components in patients with higher early energy adequacy. However, this finding should be interpreted with caution. The CALLY index was originally developed primarily in oncologic settings, and its construct validity in septic ICU populations remains uncertain. Although recent studies have suggested that CALLY may be associated with mortality and adverse clinical outcomes in sepsis patients (34, 35), in the present study it should be regarded as an exploratory composite indicator rather than a validated sepsis-specific marker. Similarly, PNI reflects both nutritional and immune-related components through albumin and lymphocyte values. Moreover, PNI and CALLY share albumin and lymphocyte components, while CRP is additionally incorporated into CALLY; therefore, these indices should not be interpreted as fully independent prognostic signals. The between-group difference observed in PNI may therefore indicate a possible association between enteral energy adequacy and short-term immune-nutritional status, although this interpretation should also remain cautious. The decrease in albumin levels observed in both groups is consistent with the well-known behavior of albumin as a negative acute-phase reactant in sepsis and may also reflect capillary leakage and hemodilution. Therefore, the early decline in albumin should be interpreted within the biological context of sepsis rather than as an isolated indicator of nutritional response.

With regard to the cellular immune response, the lower baseline lymphocyte count in the low-energy adequacy group and the subsequent increase over time should also be interpreted cautiously. Lymphocyte dynamics in sepsis are heterogeneous and may vary according to the timing of measurement, disease severity, and individual immune status; therefore, these findings cannot be directly interpreted as uniform evidence of immune recovery. Rather, they may reflect short-term temporal changes in immune-cell profiles within a complex septic response. Although experimental and clinical studies suggest that early enteral nutrition may influence immune regulation (36), the present findings do not allow a direct mechanistic inference. Likewise, the absence of a clear between-group difference in NLR over the short follow-up period may reflect the multifactorial nature of this marker in sepsis, which can limit its sensitivity for capturing the isolated contribution of nutritional adequacy.

## 5 Limitations and strengths of the study

This study has certain limitations. Firstly, this is a single-center, observational cohort study; therefore, it is not possible to establish a causal relationship. Although the nutritional interventions followed a standard protocol, this study is not a randomized controlled trial. Second, the follow-up period was limited to 7 days, and major patient-centered outcomes, including mortality, ICU length of stay, and duration of mechanical ventilation, were not assessed. Therefore, the clinical significance of the observed biomarker associations remains uncertain. Third, SOFA and APACHE II scores, septic shock status, vasopressor use, mechanical ventilation, and renal replacement therapy were unavailable and could not be included in the adjusted analyses. Although the models were adjusted for NRS-2002 and other available covariates, NRS-2002 cannot substitute for validated severity-of-illness scores. Therefore, residual confounding by disease severity and early clinical evolution remains possible. Moreover, detailed information regarding antimicrobial treatment regimens and corticosteroid exposure was also unavailable. However, the study also has significant strengths. Thanks to its prospective design, inflammatory and biochemical markers, as well as composite indices such as PNI/CALLY, were monitored in a standardized manner from days 0 to 7, and trends in changes could be reliably assessed. Furthermore, the quantitative classification of energy adequacy and the adjustment of analyses for baseline values and key covariates (age, sex, BMI, NRS-2002) significantly strengthened the internal validity of the findings, despite the observational design.

## 6 Conclusion and clinical implications

In critically ill patients with sepsis receiving early enteral nutrition, achieving ≥70% of estimated energy requirements was independently associated with lower Day 7 CRP and urea values after adjustment for baseline measurements and available covariates, suggesting a more favorable short-term inflammatory–metabolic profile. Although the prespecified between-group difference in CRP change did not reach statistical significance, the adjusted CRP and urea findings provide a clinically relevant basis for further investigation of early nutritional adequacy in sepsis. Future multicenter prospective studies incorporating standardized illness-severity scores, acute kidney injury, organ-support requirements, feeding tolerance, and patient-centered outcomes are needed to determine whether these biomarker associations translate into improved clinical outcomes and to define optimal energy and protein targets.

## Data Availability

The raw data supporting the conclusions of this article will be made available by the authors, without undue reservation.
